# Structural and electrostatic effects at the surfaces of size- and charge-selected aqueous nanodrops†
†Electronic supplementary information (ESI) available. See DOI: 10.1039/c7sc00481h



**DOI:** 10.1039/c7sc00481h

**Published:** 2017-05-19

**Authors:** Richard J. Cooper, Jeremy T. O'Brien, Terrence M. Chang, Evan R. Williams

**Affiliations:** a Department of Chemistry , University of California , Berkeley , California 94720-1460 , USA . Email: erw@berkeley.edu ; Tel: +1 510 643 7161

## Abstract

The effects of ion charge, polarity and size on the surface morphology of size-selected aqueous nanodrops containing a single ion and up to 550 water molecules are investigated with infrared photodissociation (IRPD) spectroscopy and theory. IRPD spectra of M(H_2_O)_*n*_ where M = La^3+^, Ca^2+^, Na^+^, Li^+^, I^–^, SO_4_^2–^ and supporting molecular dynamics simulations indicate that strong interactions between multiply charged ions and water molecules can disrupt optimal hydrogen bonding (H-bonding) at the nanodrop surface. The IRPD spectra also reveal that “free” OH stretching frequencies of surface-bound water molecules are highly sensitive to the ion's identity and the OH bond's local H-bond environment. The measured frequency shifts are qualitatively reproduced by a computationally inexpensive point-charge model that shows the frequency shifts are consistent with a Stark shift from the ion's electric field. For multiply charged cations, pronounced Stark shifting is observed for clusters containing ∼100 or fewer water molecules. This is attributed to ion-induced solvent patterning that extends to the nanodrop surface, and serves as a spectroscopic signature for a cation's ability to influence the H-bond network of water located remotely from the ion. The Stark shifts measured for the larger nanodrops are extrapolated to infinite dilution to obtain the free OH stretching frequency of a surface-bound water molecule at the bulk air–water interface (3696.5–3701.0 cm^–1^), well within the relatively wide range of values obtained from SFG measurements. These cluster measurements also indicate that surface curvature effects can influence the free OH stretching frequency, and that even nanodrops without an ion have a surface potential that depends on cluster size.

## Abstract 2

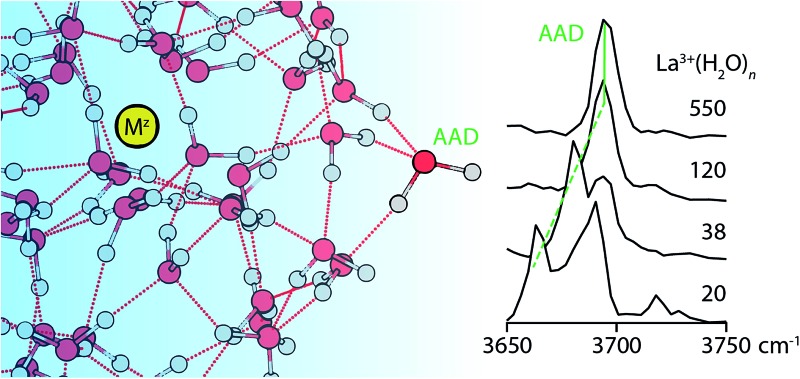
The effects of ion charge, polarity and size on the surface morphology of size-selected aqueous nanodrops containing a single ion and up to 550 water molecules are investigated with infrared photodissociation (IRPD) spectroscopy and theory.

## Introduction

Hydrated ions are ubiquitous in nature and play fundamental roles in biological and environmental phenomena. In confined nanoscale environments, interactions between ions and water molecules influence complex physicochemical processes. For example, ion channel proteins that regulate signal transduction in cells transport specific ions through cell membranes and this selectivity is based upon the size of the hydrated ion.^1–3^ In the atmosphere, ion-mediated particle nucleation is a major pathway to aerosol formation.^4–8^ The electrostatic forces between ions, water vapor, and other gaseous species stabilize small embryonic particles with diameters between 1–2 nm that seed the growth of aerosols which influence the climate^9,10^ and human health.^11,12^ Ion hydration also dictates many of the fundamental properties of aqueous electrolyte solutions. Perhaps the most well known example is the Hofmeister series where ions are ranked by their ability to precipitate out proteins in solution. Hofmeister orderings of ions have also been implicated in other solution phenomena, including salt solubilities^13,14^ and the aqueous surface activity of ions.^15–18^ Although the first reports of this ordering are over 120 years old,^19^ the molecular origins of the Hofmeister series remain debated. The effects of ions on protein stability have been attributed to direct ion–protein interactions^13,20–24^ as well as ion–water interactions that can alter the H-bonding network of water,^25–31^ and it is unclear to what extent these proposed mechanisms contribute to the observed phenomena. Thus, an understanding of how ions affect the H-bonding network of water molecules is a prerequisite for rationalizing Hofmeister effects.

While there is consensus that ions can significantly alter the structure and dynamics of water molecules in the first hydration shell from those in pure water, the spatial extent of this effect remains contentiously debated. Reports based on femtosecond infrared,^32–34^ X-ray absorption,^35,36^ and static vibrational spectroscopy^37,38^ experiments indicate that ions do not significantly affect the H-bond network of water molecules outside the first hydration shell. In contrast, results from dielectric relaxation spectroscopy,^39^ 2D IR,^40^ and X-ray scattering^41^ measurements suggest perturbations of solvent structure and dynamics that extend into at least the second shell. Directly comparing the results of these studies is complicated by the wide ranges of concentrations employed (∼0.5–6 M) as well as the obfuscating presence of counterions.^35^ Information about how ions affect the H-bond structure of water at the air–water interface has been obtained by comparing sum-frequency generation (SFG) spectra of pure water^42–45^ and ionic solutions.^46–51^ The signal intensity in SFG spectra of aqueous salt solutions changes with the identity and concentration of the dissolved ions, from which information about how ions affect interfacial water can be deduced. A joint experimental and theoretical study of aqueous ammonium sulfate and ammonium chloride solutions found that the structure of interfacial water depends strongly upon the charge density of the anion. The SFG signal of aqueous (NH_4_)_2_SO_4_ solutions is greater than that of NH_4_Cl solutions.^49^ This enhancement in signal was attributed to differences in the hydration of SO_4_^2–^ and Cl^–^, where the strongly hydrated sulfate ion lies at one extreme end of the Hofmeister series. MD simulations indicate that SO_4_^2–^ strongly interacts with water molecules in solution and is consequently depleted from the interface. This has the net effect of increasing the depth of the interfacial region, leading to an enhancement in SFG signal. In contrast, the comparatively weakly hydrated ion Cl^–^ is present at the interface in concentrations similar to that in bulk. SFG measurements indicate that the larger, more polarizable halides have enhanced concentrations at air–water interfaces, linking the Hofmeister behavior of these ions to their surface activity.^52^ These studies demonstrate that intrinsic ion–water interactions can lead to substantial changes in the structure of aqueous interfaces.

An alternative approach to studying ion hydration is through measurements on gaseous hydrated ions where counterion effects can be studied explicitly^53,54^ or can be eliminated. The effect of a single ion or electron on water structure can be investigated as a function of hydration extent, ranging from a single water molecule up to hundreds of water molecules, and information about ion solvation can be obtained in the limit of infinite dilution.^55–61^ Infrared photodissociation (IRPD) spectroscopy can probe the structures of numerous gas-phase hydrates, including metal ions,^62–68^ anions^26,28,69–72^ and protonated/ionized amino acids and peptides.^73–76^ Heteroatom-hydrogen stretching frequencies are sensitive to their local H-bonding environment, and IRPD measurements have been used to elucidate detailed hydration structures of ions with up to ∼20 water molecules attached.^77,78^ IRPD spectra measured under readily achievable experimental conditions can be compared to calculated absorption spectra of candidate structures to identify populations of distinct isomers in the experimental ensemble.^79–81^ For more extensively hydrated ions, identifying single isomers is generally not feasible, but information about how ions affect the H-bonding network of water in nanometer-sized droplets can still be deduced from spectral signatures. On account of the large surface area-to-volume ratios of these nanodrops, their IRPD spectra contain sharp resonances from surface water molecules with hydrogen atoms that do not participate in H-bonding (“free” OH bonds) similar to, but significantly narrower than, those observed in SFG spectra at the air–water interface.^42–45^ Free OH stretching frequencies (typically ∼3650–3750 cm^–1^) are sensitive to the H-bonding environment of the parent water molecule as well as the electric field at a nanodrop's surface. Evidence that the multiply charged ions SO_4_^2–^, Fe(CN)_6_^3–^ and Fe(CN)_6_^4–^ can perturb the structure of water molecules into the second and third solvation shells has been deduced from IRPD spectra in the free OH region.^25,26,28^ Results from IRPD spectroscopy of hydrated ions at fixed cluster size (*n* ∼ 36 and *n* ∼ 250) indicate that ions can affect the water molecules at the surface of the nanodrop.^27,82^ The free OH stretch frequencies in these clusters depend on the electric field of the ion, and this Stark shift has been used to deduce the stretching frequencies of free OH oscillators in corresponding neutral droplets. However, a fully size-resolved investigation of how nanodrop structure evolves as a function of cluster size, ion charge state, and ion polarity has not been previously reported.

Here, we present results from IRPD spectroscopy in the free OH stretch region of M(H_2_O)_*n*_ where M = La^3+^, Ca^2+^, Na^+^, Li^+^, I^–^, and SO_4_^2–^ and *n* ranges from 20 water molecules up to a maximum of 550 water molecules, recorded at 133 K. The spectra show that ion charge state and polarity strongly influence H-bonding motifs of water molecules at the surfaces of the smaller nanodrops, consistent with results from supporting MD simulations. For the multivalent cations, there are pronounced Stark shifts for free OH stretches of hydrates with less than ∼100 water molecules, which we attribute to the ion perturbing the H-bonding network of water molecules that extends to and affects the orientation of water at the surface of the nanodrop. These size-resolved measurements reveal surface curvature effects in smaller droplets that provide insights into the inherent structure of surface water in confined environments. A precise value for the free OH stretch frequency of a neutral water molecule at the surface of water is obtained by extrapolating these data to infinite dilution.

## Experimental methods

### IRPD spectroscopy

Experiments were performed using a 7.0 T Fourier transform ion cyclotron resonance (FT-ICR) mass spectrometer coupled to a tunable infrared laser. The instrument, recently upgraded from a 2.7 T magnet, is described elsewhere.^83^ Briefly, hydrated ions are formed by nanoelectrospray ionization of ∼5 mM solutions of LaCl_3_, CaCl_2_, NaCl, KI, (Sigma-Aldrich, Saint-Louis, MO) and CuSO_4_ (Fischer Scientific, Fair Lawn, NJ) dissolved in ultrapure water (Millipore, Billerica, MA). The hydrated ions are guided by electrostatic lenses through five stages of differential pumping into the FT-ICR cell. A pulse of dry nitrogen gas (∼10^–6^ Torr) is introduced into the high vacuum chamber through a piezoelectric valve to aid with the trapping and thermalization of the ions to the temperature of a copper jacket^84^ surrounding the cell. The jacket is maintained at a temperature of 133 K by a regulated flow of liquid nitrogen. After accumulating ions for 5–7 s, there is a pump down delay of similar length to allow the pressure in the cell to return to <10^–8^ Torr. Precursor ions are selected using stored waveform inverse Fourier transforms. An ensemble averaging technique^85^ is used for improving the signal-to-noise ratios of the spectra by mass selecting multiple precursor ions that have adjacent hydration states. For small clusters containing less than ∼150 water molecules, ensembles containing 1–3 precursor ions are selected whereas for 150 ≤ *n* < 300 and 300 ≤ *n* ≤ 550, ensembles containing 3–5 and 7–11 neighboring cluster sizes are used, respectively. A weighted-average cluster size is reported throughout.

Infrared action spectra are acquired by measuring the average cluster size of a group of mass-selected ions before and after laser irradiation. The mass-selected precursor ions are irradiated at specific frequencies between 3650–3750 cm^–1^ with infrared light from an OPO/OPA tabletop laser system (LaserVision, Bellevue, WA) pumped by the 1064 nm fundamental of a Nd:YAG laser (Continuum Surelight I-10, Santa Clara, CA) at a 10 Hz repetition rate. Irradiation times between 0.5–2 s are chosen to induce substantial, but not complete, fragmentation of the precursor ions that dissociate by the sequential loss of single water molecules. A first-order dissociation rate constant is obtained from the extent of water loss and the duration of the irradiation. Because these clusters have sufficient internal energies to dissociate under ambient conditions in the ion trap, a blackbody infrared radiative dissociation (BIRD) rate constant is also measured in the absence of laser irradiation.^85^ The reported IRPD rate constant is then determined by subtracting the BIRD rate constant from the rate of water loss with the laser on; in this way, only dissociation induced by the laser is included in the IRPD rate constant. IRPD rate constants are also corrected for frequency-dependent variations in laser power.

### Computational chemistry

Molecular dynamics (MD) simulations of (H_2_O)_*n*_ and M(H_2_O)_*n*_ where M = Mo^3+^, Ca^2+^, Na^+^, I^–^ and SO_4_^2–^ and 20 ≤ *n* ≤ 300 were performed using the OPLS_2005 force field implemented in MacroModel 11.2 (Schrödinger, Inc., Portland, OR). After an initial geometry relaxation with molecular mechanics, the systems were allowed to equilibrate for 10 ns using canonical ensemble stochastic dynamics at 133 K. Integration time steps of 1.5 fs were used and the SHAKE algorithm was applied to all hydrogen atoms. The final equilibrated geometries were used to run 50 ns dynamics trajectories, from which 1000 structures were saved for each ion. Evaporative water loss was not observed in any of the dynamics trajectories because they are run for tens of nanoseconds, whereas experimentally the timescale for dissociation is milliseconds to seconds. An in-house routine written in MATLAB (The Mathworks, Inc., Natick, MA) was used to characterize the H-bonding environments within the identified structures. From the saved *xyz* coordinates, O–H···O distances and angles were calculated for every OH bond in a given structure in order to discern free and H-bonded OH stretches. The distance cutoff for a H-bond was chosen to be 2.6 Å, and the maximum angular deviation of the O–H···O angle from linearity was set to 60°. Identified free OH bonds were categorized according to the H-bonding environment of the parent water molecule based on the number of H-bonds it accepted and donated. For the M(H_2_O)_*n*_ structures where *n* = 250, the program was also used to calculate the angle *θ* between the dipole vector of each water molecule located at a distance *d* from the ion and the radial vector defined by the ion-oxygen displacement. In the case of the polyatomic ion SO_4_^2–^, the location of the ion was chosen to be its center of mass. Similar data was also calculated for (H_2_O)_250_ clusters using the center of mass to calculate the radial vector. The resulting distance and angle data (*d*, *θ*) from each of the 1000 structures in three trajectories was binned in 0.5 Å increments, and the average angle *θ* in each bin was calculated.

At present, infrared spectra calculated from *ab initio* methods are prohibitively expensive for the majority of cluster sizes investigated here. An alternative approach is to calculate OH stretch spectra directly from cluster geometries identified by molecular dynamics trajectories.^38,86,87^ This approach, originally devised to calculate the vibrational spectrum of bulk water, is based on the proportionality between the frequency shift of an OH oscillator and the component of the local electric field projected along the OH bond vector. For each of the 1000 structures identified for a given cluster, each hydrogen and oxygen atom is assigned a charge of +0.41 and –0.82*e*, respectively, and the ion is assigned its formal charge. From these assigned point charges, the electric field at each hydrogen atom H_i_ arising from the ion and all the hydrogen and oxygen atoms of *other* water molecules in the cluster is calculated. The resulting electric field vector at each H_i_ is then projected along the OH_i_ bond unit vector to yield the component of the field along the bond, *E*_i_, in atomic units. Local OH stretch frequencies *f*_i_ (in cm^–1^) are calculated from the mapping *f*_i_ = 3762 – 5060 × *E*_i_ – 86 225 × *E*_i_^2^ where the coefficients are empirical parameters determined by fitting frequencies from *ab initio* calculations on neutral water clusters.^86,87^ Other parameters, such as intramolecular coupling constants and frequency dependent transition dipole moments have also been parameterized in terms of the electric field at a given H atom.^86^ For each water molecule, we obtain coupled OH stretching frequencies from the uncoupled OH stretch frequencies by solving for the eigenvalues of the two-state system where the off-diagonal elements are the coupling constants given in ref. 86. The distribution of absorption frequencies is plotted as a histogram of the coupled OH frequencies from all 1000 structures. Relative infrared intensities are calculated by multiplying the histograms bin counts by the square magnitude of the frequency-dependent transition dipole moment provided in ref. 86.

## Results and discussion

### Spectral progression of free OH bands

**Fig. 1:**
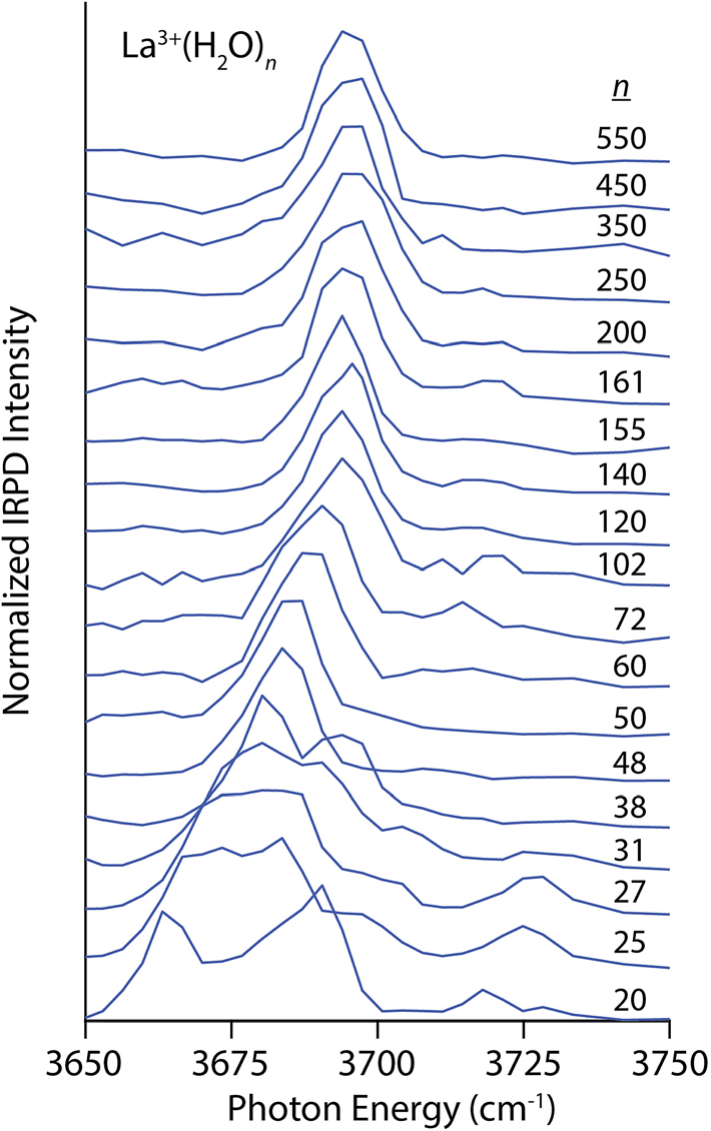
IRPD spectra of La^3+^(H_2_O)_*n*_ for 20 ≤ *n* ≤ 550 measured at 133 K.

**Fig. 2:**
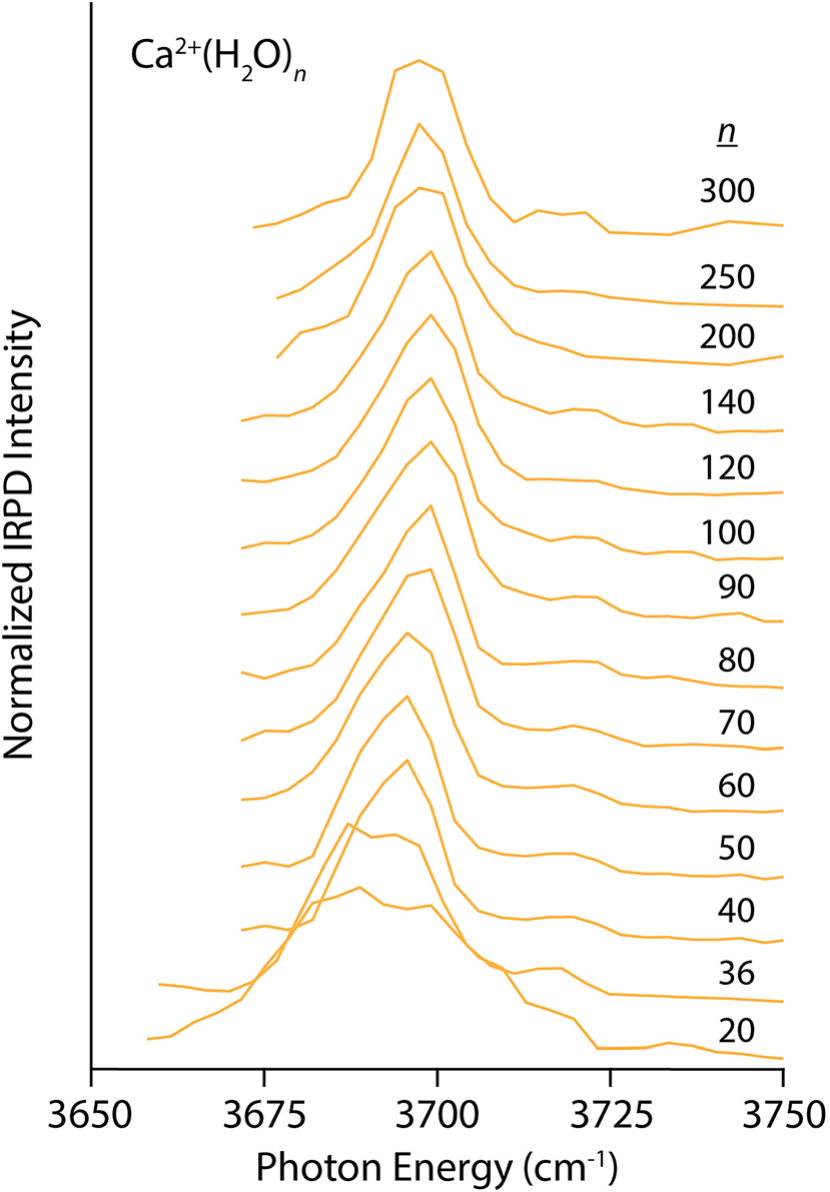
IRPD spectra of Ca^2+^(H_2_O)_*n*_ for 20 ≤ *n* ≤ 300 measured at 133 K.

**Fig. 3:**
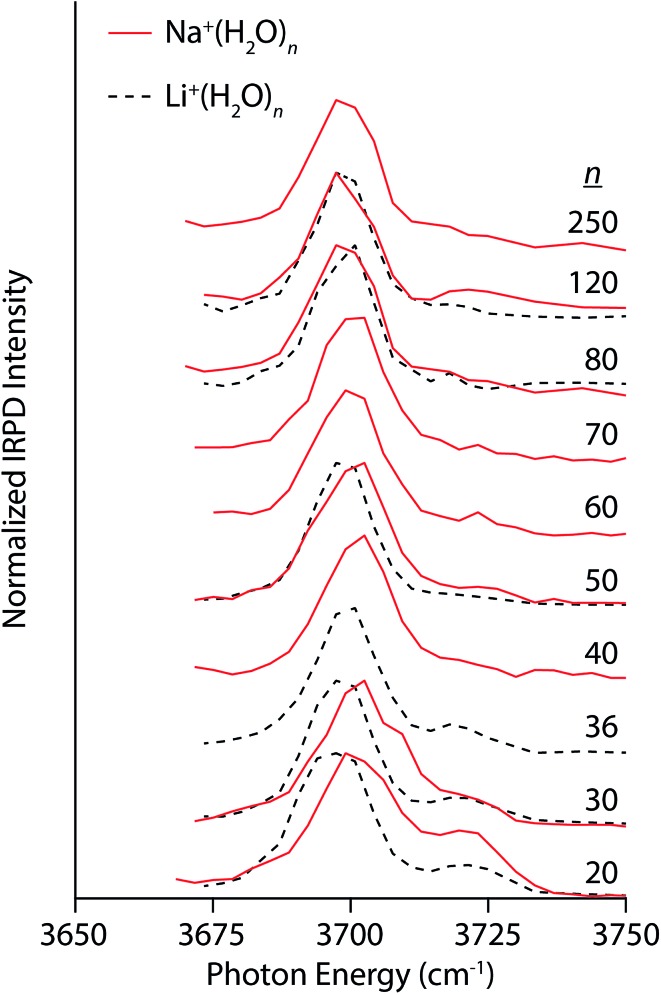
IRPD spectra of Na^+^(H_2_O)_*n*_ (solid red lines) and Li^+^(H_2_O)_*n*_ (dashed black lines) for 20 ≤ *n* ≤ 250 and 20 ≤ *n* ≤ 120, respectively, measured at 133 K.

**Fig. 4:**
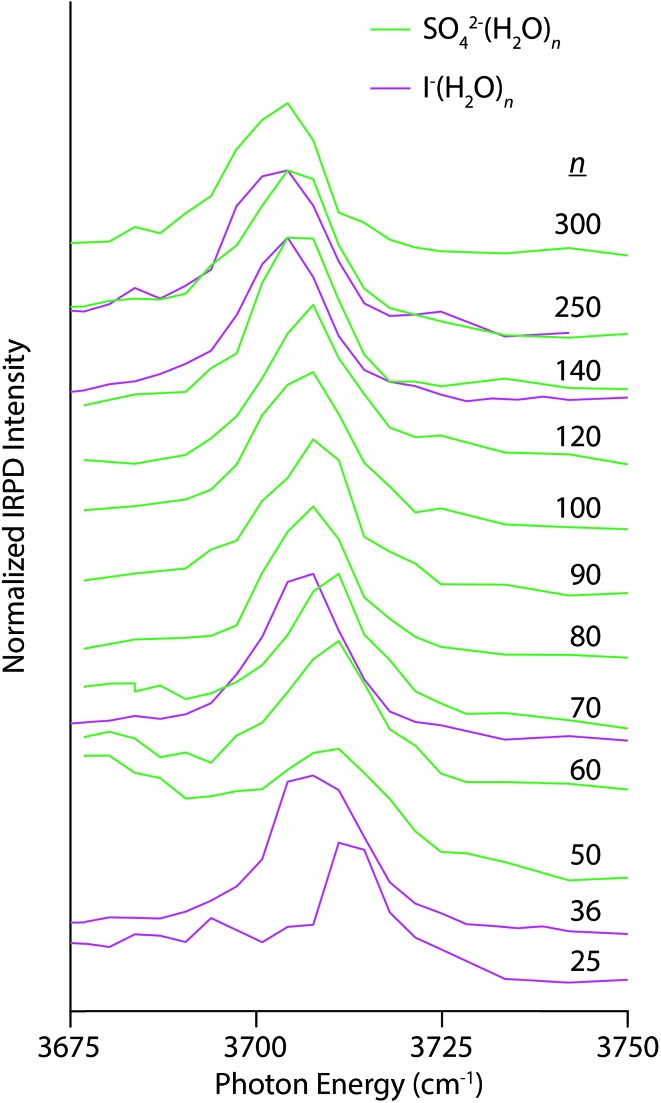
IRPD spectra of SO_4_^2–^(H_2_O)_*n*_ (solid green lines) and I^–^(H_2_O)_*n*_ (solid magenta lines) for 50 ≤ *n* ≤ 250 and 25 ≤ *n* ≤ 250, respectively, measured at 133 K.

Photodissociation spectra in the free OH stretch region (3650–3750 cm^–1^) of size-selected clusters of M(H_2_O)_*n*_, where M = La^3+^, Ca^2+^, Na^+^, Li^+^, I^–^, and SO_4_^2–^ are shown in Fig. 1–4. The interpretation of these spectra is guided by two principles. First, the number of distinguishable bands is related to the heterogeneity of H-bonding environments of water molecules at the surfaces of these clusters. The frequencies of free OH stretches are exquisitely sensitive to a water molecule's H-bonding environment,^62–68,79,88,89^ and the free OH stretch of a water molecule red-shifts as the water molecule's participation in H-bonding increases. Using the nomenclature where “A” denotes a H-bond accepted by a water molecule and “D” denotes a H-bond donated by a water molecule, free OH bands appear in increasing frequency following the order AAD < AD ≈ AA < A. This ordering refers to antisymmetric free OH bands that are significantly more intense and sharper than the corresponding symmetric stretches and thus more useful for structural characterization.^79^ Second, the frequencies of these bands are affected by the charge, size and polarity of the ion^27,82,90^ as well as the proximity of the OH oscillator to the ion.^82^ Highly charged cations induce the greatest red-shift whereas anions cause these stretches to blue-shift. For ions that have the same charge state but differ in size, the magnitude of the Stark shift is greatest for the smaller ions.^82^

The spectra of La^3+^(H_2_O)_*n*_ clusters for 20 ≤ *n* ≤ 550 exhibit significant changes in both H-bonding motifs and Stark shifting as a function of cluster size (Fig. 1). For the smallest cluster, La^3+^(H_2_O)_20_, three distinct bands appear in the spectrum centered on 3665 cm^–1^, 3685 cm^–1^ and 3720 cm^–1^. This spectrum strongly resembles previously reported IRPD spectra of La^3+^(H_2_O)_17–20_,^91^ from which the lowest-energy band is assigned to AAD stretches whereas the broader band from ∼3675–3700 cm^–1^ arises from AD and AA stretches that have similar frequencies at this cluster size. IRPD rate constants in the free OH region can be measured with a high level of reproducibility; a previous study has shown that relative standard deviations in these measurements are within ±5%.^92^ The highest energy band reflects a population of under-coordinated A water molecules, indicating that H-bonding between water molecules is not optimized in this cluster on account of strong interactions with La^3+^ that orient water molecules in the interior resulting in sub-optimal H-bonding at the surface. At *n* = 25, the splitting between the lower energy bands decreases resulting in one broad resonance between 3660–3680 cm^–1^ with a small shoulder at 3700 cm^–1^. The high energy A band near 3725 cm^–1^ is still clearly visible and is, in fact, slightly more intense. With increasing cluster size, the spectra of these La^3+^ hydrates simplify, indicating the onset of more optimal H-bonding between water molecules. All bands in the free OH region exhibit a blue-shift with increasing cluster size as the surface water molecules become more remote from the solvated ion. The band for dangling A water molecules disappears by *n* = 38 and on the low energy side, the AAD band dominates the spectrum by *n* = 48. For larger clusters, the spectra consist of an intense AAD band with a much weaker band lying about 20 cm^–1^ higher in frequency at some cluster sizes. This band reflects a minor population of AD water molecules at the nanodrop surface, and for many cluster sizes, this band is just above the baseline of the spectrum. There is no evidence of an AD band in the spectra of the largest La^3+^ hydrates (*n* = 500 and 550), suggesting more optimal H-bonding at the surfaces of these clusters. The Stark shift of the AAD band is particularly striking in these spectra; there is ∼30 cm^–1^ red-shift going from *n* = 102 to *n* = 20. This signals that the electric field of La^3+^ strongly perturbs the frequencies of free OH stretches located remotely from the ion. The Stark shift with increasing cluster size is more gradual for larger clusters, and is on the order of only a few cm^–1^.

Similar trends, although less pronounced, are apparent in the IRPD spectra of Ca^2+^(H_2_O)_*n*_ for *n* between 20 and 300 (Fig. 2). The spectrum of the *n* = 20 cluster contains one broad resonance spanning 3660–3720 cm^–1^ that encompasses AAD, AD, and AA stretches. There is very weak dissociation in the region between 3725–3750 cm^–1^ consistent with a minor population of A water molecules, but clearly not to the extent observed in the small La^3+^ hydrates. This indicates that water–water interactions are more optimal in Ca^2+^ hydrates than La^3+^ hydrates, as one might expect based on the lower charge of the ion. The spectra quickly simplify with increasing cluster size and contain a dominant AAD band as well as a small AD band. The Stark shift of the AAD band is ∼10 cm^–1^ for *n* between 20 and 100, significantly smaller than the 30 cm^–1^ Stark shift for the La^3+^ hydrates. This difference in the magnitude of the Stark shift is consistent with the charge state of the ion being a primary factor, but other factors, such as solvent structuring, charge transfer and ion size also contribute to this effect.

Compared to the multivalent cations, the IRPD spectra of Na^+^(H_2_O)_*n*_ and Li^+^(H_2_O)_*n*_ show different size-dependent changes (Fig. 3). For Na^+^(H_2_O)_20_, there are two distinct bands centered on 3701 cm^–1^ and 3720 cm^–1^ corresponding to AAD and AD water molecules, respectively. The AD band decreases with cluster size, becoming only a small shoulder in spectra of clusters larger than *n* = 30. Interestingly, the AAD band blue-shifts with decreasing cluster size in striking contrast to the trends observed in the spectra of La^3+^ and Ca^2+^ hydrates. The magnitude of this Stark shift is quite small: approximately 4 cm^–1^ between *n* = 30 and 250. The simple band structure in these spectra signify that Na^+^ weakly perturbs the H-bonding structure of water. Thus, the blue-shifting of the AAD band with decreasing cluster size indicates that there is another size-dependent electric field at the surface of the nanodrops in addition to that of the ion. This surface electric field is likely established by the intrinsic structure of water in these small nanodrops and depends on the curvature of the surface. Spectra of Li^+^(H_2_O)_*n*_ were also recorded to investigate how an ion's size affects Stark shifting and droplet structure. Li^+^ has an ionic radius of 90 pm compared to 116 pm for Na^+^. The lower intensity of the AD band in the spectrum Li^+^(H_2_O)_20_ relative to that of Na^+^(H_2_O)_20_ is on account of Li^+^ forming partial clathrate structures at this “magic number” cluster size.^78^ The AAD band in the spectra of Li^+^(H_2_O)_*n*_ for *n* = 20 and 30 is red-shifted by ∼4 cm^–1^ compared to the corresponding Na^+^ spectra, consistent with the smaller ionic radius and higher charge density of Li^+^. Differences between the two singly charged ions diminish with increasing cluster size; the band frequencies differ by ∼2 cm^–1^ for *n* = 50 and <1 cm^–1^ for *n* = 80 and 120. Thus, the identity of an ion can affect droplet structure and Stark shifting particularly at small cluster sizes and these effects become less pronounced with increasing cluster size.

Electrostatic interactions between anions and solvating water molecules lead to inherently different hydration motifs in anionic clusters, which are manifest in the IRPD spectra of SO_4_^2–^(H_2_O)_*n*_ and I^–^(H_2_O)_*n*_ (Fig. 4). For example, the spectral progression for SO_4_^2–^(H_2_O)_*n*_ begins at *n* = 50 because clusters smaller than *n* ∼ 47 do not contain a free OH band in their IRPD spectra at this temperature.^28^ Water molecules in these clusters have both OH bonds oriented inwards towards the sulfate ion thereby establishing a H-bonding network that is oriented in the opposite direction to the hydration motif for nanodrops containing cations wherein the OH bonds are directed outwards away from the solvated ion. In fact, the appearance of a free OH stretching band in photodissociation spectra of hydrated multiply charged anions has been taken as a metric of the spatial extent of ion-induced patterning of the H-bond network.^25,26,28^ This structuring effect can extend into the third solvation shell and beyond. The AAD band in the spectrum of SO_4_^2–^(H_2_O)_50_ appears as a broad band superimposed on the high-energy tail of dissociation from H-bonded OH stretches and grows in relative intensity with increasing cluster size. The Stark shift of this band with decreasing cluster size is clearly in the opposite direction of the multivalent cations and is similar in magnitude to the Ca^2+^(H_2_O)_*n*_ clusters for 50 ≤ *n* ≤ 300 (4 cm^–1^ for Ca^2+^ and 6 cm^–1^ for SO_4_^2–^). A similar trend is observed in the IRPD spectra of I^–^(H_2_O)_*n*_, although for this singly charged anion, there is already a free OH band by *n* = 25 owing to weaker ion–water interactions. At common cluster sizes where spectra were recorded (*n* = 70, 140, and 250), the blue-shift in the free OH band of I^–^(H_2_O)_*n*_ is less than that of SO_4_^2–^(H_2_O)_*n*_, consistent with the lower charge of the former ion.

### Stark shifting of surface OH stretches

**Fig. 5:**
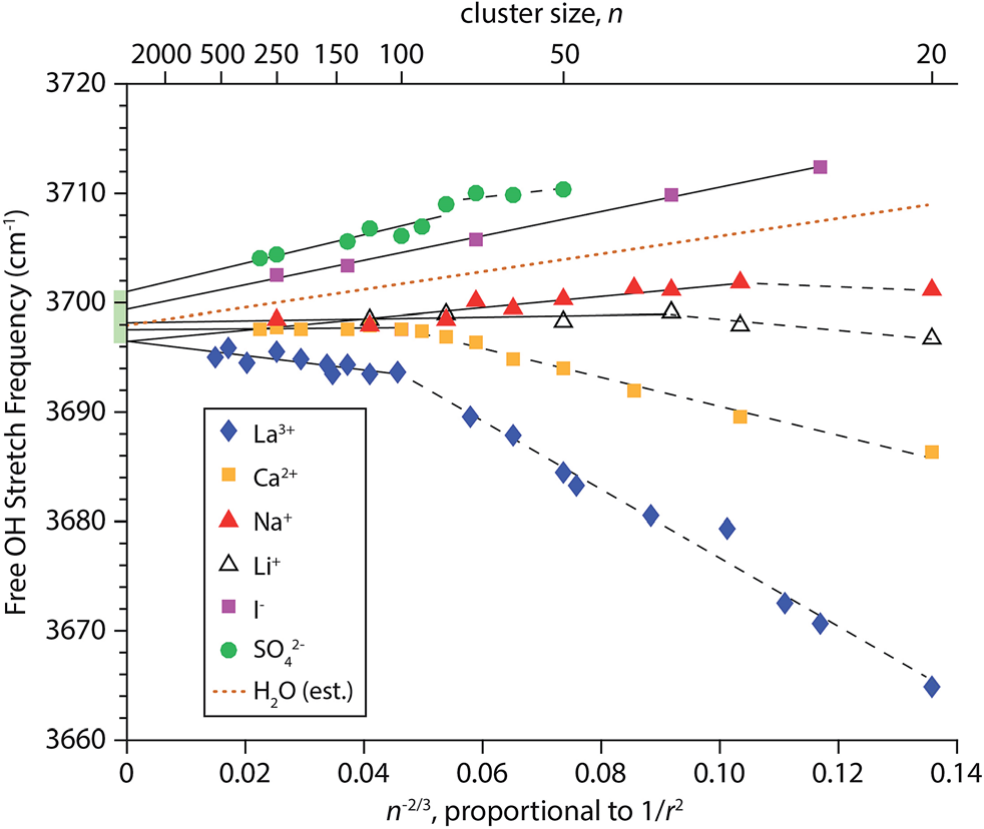
Fitted centroid frequencies of the AAD free OH bands for M(H_2_O)_*n*_ where M = La^3+^, Ca^2+^, Na^+^, Li^+^, I^–^ and SO_4_^2–^ as a function of *n* ^–2/3^, which is proportional to 1/*r* ^2^ where *r* is the droplet radius. For ions where the observed Stark shift does not linearly depend on the ion's electric field strength, separate linear fits are shown for the larger and smaller cluster sizes as solid and dashed lines, respectively. The extrapolated range of free OH frequencies at infinite cluster size spans 4.5 cm^–1^ and is shown as a green shaded rectangle. Free OH stretching frequencies of neutral (H_2_O)_*n*_ clusters interpolated from the measured Na^+^ and I^–^ data are shown as a dotted brown line.

To obtain a better understanding of how the frequencies of surface-bound free OH stretches change with cluster size, the AAD bands in the IRPD spectra in Fig. 1–4 were fit with Gaussian line shapes to extract the centroid of each peak. Frequency step sizes between 3 and 4 cm^–1^ were used throughout the free OH region so that each AAD band consists of 7–9 data points. The band frequencies determined from the fits have standard errors that range from 0.16 to 0.39 cm^–1^, reflecting the high precision to which these values can be measured. The resulting centroid frequencies from these fittings are shown in Fig. 5 as a function of *n* ^–2/3^, which is proportional to 1/*r* ^2^ where *r* is the radius of the droplet. This relationship between cluster size and radius holds for spherical nanodrops and there is experimental^55^ and computational^26,55,82^ evidence that indicates these nanodrops are approximately spherical. Within first-order perturbation theory, the Stark shift of an OH oscillator relative to its gas-phase value is linearly proportional to the local electric field at the H atom projected along the OH bond.^93,94^ The observed trend that free OH stretches of nanodrops containing highly charged cations redshift with decreasing cluster size (and increasing proximity to the ion) whereas nanodrops containing anions blue-shift is characteristic of Stark shifting due to the electric field of the solvated ion. However, considering that the electric field of an atomic ion drops off as 1/*r* ^2^, one might expect a linear relationship between the frequency of surface OH oscillators and the inverse square of the cluster radius. Our results indicate that this is not necessarily the case; the measured shifts of free OH stretches exhibit varying degrees of non-linear behavior as a function of 1/*r* ^2^. This is especially apparent for hydrates of the multiply charged cations La^3+^ and Ca^2+^. For these ions, it appears that there are two regimes delimited by cluster size. Pronounced shifting is observed for the smaller clusters (*n* < ∼100) followed by a transition to a regime where the shifts are significantly reduced in magnitude.

For each ion besides iodide, an optimal least-squares fitting of the frequency data in Fig. 5 using two lines was performed. The data for iodide are highly linear (*R* ^2^ = 0.997) and therefore only a single line was used for the fitting. In order to select the cluster size marking the transition between the two regimes in an unbiased way, a MATLAB script was written that evaluates all possible ways of fitting the frequency data for a given ion with two lines. The best fit was chosen to be the one that gave the smallest residuals. For sulfate, fitting the data with two lines *versus* a single line leads to only a slightly improved fit, with calculated root-mean-square residuals of 0.49 cm^–1^ *versus* 0.63 cm^–1^, respectively. These linear fittings are shown in Fig. 5 where the break in the fittings is denoted by a dashed line for smaller cluster sizes and a solid line for larger cluster sizes. The identified transition cluster size is correlated to the ion's charge (*n* = 102 for La^3+^, *n* = 100 and 80 for Ca^2+^ and SO_4_^2–^, respectively, and *n* = 36 and 30 for Li^+^ and Na^+^, respectively). This suggests that the different frequency shifting regimes are related to the ion's ability to affect the orientations of water molecules at the surfaces of these nanodrops. Water is a polarizable medium with a large molecular dipole moment and its structure is thus susceptible to the influence of an ion's electric field. This patterning effect can propagate to the surface of a nanodrop thereby affecting the orientations of free OH bonds at the surface, as has been demonstrated by IRPD studies on multiply charged anions.^25,26,28^ Free OH bonds that are better aligned with the radial electric field of the ion will have a greater Stark shift owing to the improved overlap, as is the case for La^3+^ and Ca^2+^ hydrates at small cluster size. Conversely, in nanodrops where there is poorer alignment between free OH bonds and the ion's electric field, the magnitude of the Stark shift will be smaller. This may explain why the measured Stark shifts in smaller SO_4_^2–^(H_2_O)_*n*_ clusters are less pronounced; structures obtained from MD simulations indicate that free OH bonds in these clusters are directed more tangentially to the nanodrop's surface (*vide infra*). Interestingly, the frequency data for La^3+^(H_2_O)_*n*_ transitions at *n* = 102 and it has previously been reported that the onset of crystallinity in size-selected La^3+^ – doped aqueous nanodrops is delayed by ∼100 water molecules compared to neutral water clusters.^55^ The frustration of crystallinity was attributed La^3+^ disrupting optimal H-bonding of water molecules located remotely from the ion. Our present results for La^3+^ are consistent with this finding and indicate a relationship between the extent of Stark shifting and the ability of an ion to perturb the H-bonding network of water.

The precise control of nanodrop size and charge made possible by mass-selection also precludes measurements on neutral droplets with this technique. However, some properties of neutral droplets and bulk water can be inferred from the linear fit parameters of the Stark shifting data (Table S1†). For example, the slopes of the fits to the larger clusters of the monovalent cations (*m* = 51.2 and *m* = 8.4 for Na^+^ and Li^+^, respectively) are positive, indicating a blue-shift with decreasing cluster size despite the positive charge on these ions. There is a much larger blue-shift in the slope of the linear fit to iodide (*m* = 111.6). Interpolation between the measured slopes for Na^+^ and I^–^ at large cluster size yields a positive slope (*m* = 81.4) that indicates the free OH stretches of neutral water clusters blue-shift with decreasing cluster size (Fig. 5, dotted line). Experimental evidence for a small blue-shift of the free OH band in neutral water clusters with decreasing cluster size has been reported, although the origin of this effect has not previously been explained.^95,96^ In the absence of an ion, a Stark shift could arise from electric fields generated by the intrinsic structure of interfacial water. At the bulk air–water interface, the electric field can have a non-zero component directed in the surface normal direction owing to the net dipolar orientation of water molecules. This establishes a surface potential at the interface, where a positive value indicates a net orientation of water molecule hydrogen atoms towards the bulk and a negative value indicates that water molecules tend to orient their hydrogen atoms towards the vapor phase.^97^ Experiments on dilute ionic solutions indicate that the surface potential is small and positive (∼0.1 V).^98,99^ Our Stark shift data suggest that the electric field established at the surfaces of small neutral nanodrops is more similar to anion-containing nanodrops than cation-containing nanodrops. This indicates that the molecular orientation of interfacial water molecules is such that as the neutral clusters become smaller, the surface layer gains a net orientation where hydrogen atoms are directed inward thereby creating a partial negative charge at the nanodrop surface. In this scenario, the surface potential becomes increasingly positive with decreasing cluster size, although the absolute sign of the surface potential (*i.e.* positive or negative) cannot be deduced from OH stretching data alone. Previous experiments on neutral water clusters indicate that clusters with diameters less that ∼4 nm have a reduced surface density of dangling OH oscillators,^96^ which is qualitatively consistent with this explanation. Another factor that may contribute to the observed blue-shifting is increasing strain in the H-bond network of water at the surfaces of smaller clusters, which would have the effect of weakening H-bonding between water molecules, resulting in stronger free OH bonds.

Extrapolation of the linear fits of the Stark shift data for large clusters to infinite cluster size yields AAD free OH stretch frequencies that range from 3696.5–3701.0 cm^–1^. This narrow range of frequencies obtained from larger nanodrops containing a broad range of different charge states indicates that the surfaces of the nanodrops are similar in each of these clusters and that the frequency shifts for the larger clusters are due primarily to the different charges on the ions and not due to a difference in the orientation of water at the nanodrop surface. Thus, this narrow range of frequencies should bracket the frequencies of the corresponding surface OH stretch in bulk water. Previous extrapolations of the neutral AAD free OH stretch from IRPD data of hydrated ions at fixed cluster size yielded estimates of 3704.9–3709.7 cm^–1^ for clusters with ∼36 water molecules^82^ and 3699.3–3700.1 cm^–1^ for clusters with ∼250 water molecules.^27^ Because these estimates pertain to the free OH stretches of water molecules in neutral droplets at specific cluster sizes, they include surface curvature effects. The range of frequencies reported here (3696.5–3701.0 cm^–1^) is obtained in the limit of infinite droplet size (*i.e.* no surface curvature), and is therefore the first measurement of the AAD free OH stretch frequency of neutral bulk water from IRPD spectroscopy, which under these conditions should be similar to a linear spectroscopy. This range of values agrees well with those from SFG measurements of bulk aqueous interfaces near room temperature (3690–3705 cm^–1^).^42,43,45,49,50^ The free OH band in SFG spectra appears as a broad feature (∼50 cm^–1^ fwhm) and its frequency and line shape is dependent upon the polarization and orientations of the incident laser beams resulting in a greater uncertainty in determining free OH stretching frequencies. Under the cold conditions of our experiment, the phase of water in the interior of the droplet begins to resemble crystalline ice for *n* ≥ 375.^55^ Thus, for the larger La^3+^(H_2_O)_*n*_ clusters, the free OH band should more closely correspond to bulk ice although there is no significant change in the frequency or line shape of the free OH bands for these cluster sizes. This is consistent with temperature dependent SFG studies reporting that the free OH stretch frequency is insensitive to temperature suggesting a strong resemblance between the surfaces of ice and liquid water.^43,100,101^

### Modeled Stark shifting

**Fig. 6:**
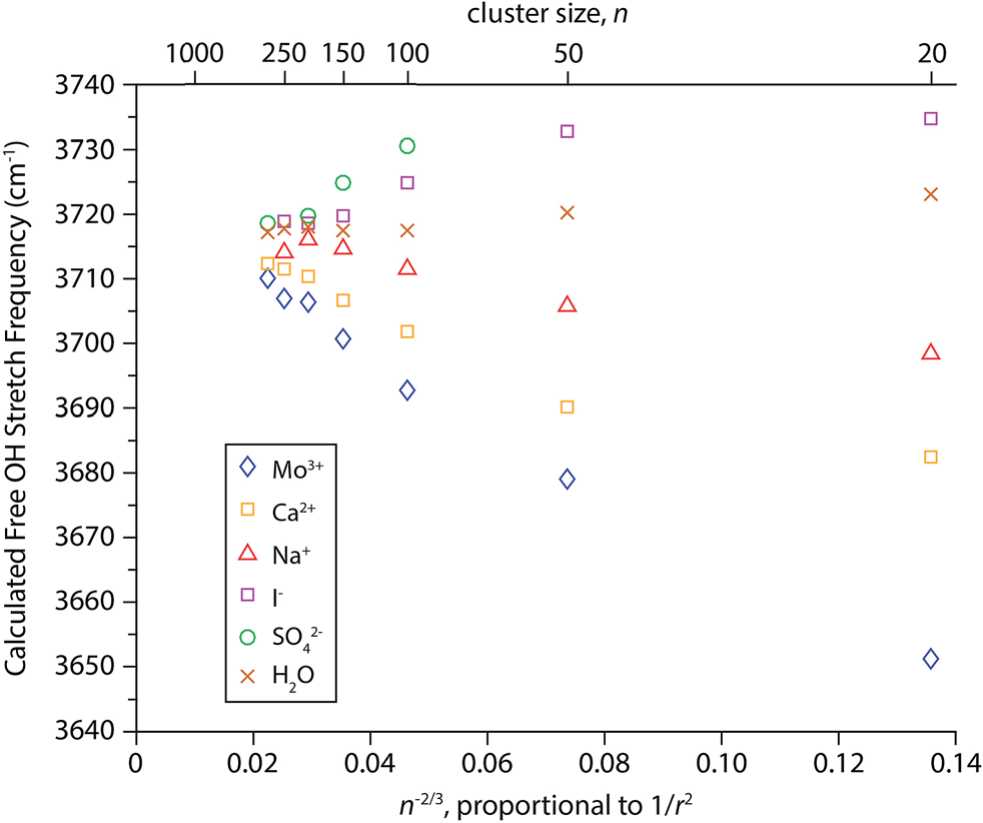
Centroid frequencies of the AAD free OH bands from simulated spectra of (H_2_O)_*n*_ and M(H_2_O)_*n*_ where M = Mo^3+^, Ca^2+^, Na^+^, I^–^ and SO_4_^2–^ as a function of *n* ^–2/3^, which is proportional to 1/*r* ^2^ where *r* is the droplet radius.

Simulated infrared spectra of M(H_2_O)_*n*_ for M = Mo^3+^, Ca^2+^, Na^+^, I^–^, and SO_4_^2–^ with *n* ranging between 20 and 300 water molecules were calculated directly from cluster geometries generated by dynamics simulations. Spectra of cluster sizes larger than *n* ∼ 300 could not be calculated due to MATLAB memory restrictions and Mo^3+^ was substituted for La^3+^ because it is the largest trivalent ion parameterized in the software used for the dynamics simulations. The electrostatic model used to calculate these spectra, originally implemented in simulations of bulk water, is based on the relationship between the stretching frequency of an OH oscillator and the local electric field it experiences.^86,87^ This model was previously adapted to calculate infrared spectra of various hydrated gaseous ions at fixed cluster size (*n* = 36).^82^ Here, we use the simulated spectra to investigate the physical origins of the frequency shifts of the free OH bands as a function of cluster size. For each cluster simulated, the resulting infrared spectra have resonances arising from both free and bonded OH stretches (see Fig. S1† for examples). The AAD bands in the free OH region were fit with a Gaussian line shape and centroid frequencies extracted from these fits are shown as a function of *n* ^–2/3^, which is proportional to 1/*r* ^2^ (Fig. 6). Results from this model reproduce several of the experimental trends. The direction of Stark shifting is clearly correlated to ion polarity with the free OH stretching frequencies of cationic clusters red-shifting and anionic clusters blue-shifting with decreasing cluster size. The free OH frequencies of the neutral water clusters change only slightly with cluster size. The smaller (H_2_O)_50_ and (H_2_O)_20_ clusters are blue-shifted by ∼3 cm^–1^ and 6 cm^–1^, respectively, compared to the larger water clusters. In ion-containing clusters, the extent of Stark shifting depends on the charge state of the ion where the largest frequency shifts are calculated for the multivalent ions (Mo^3+^, Ca^2+^, and SO_4_^2–^). These results provide further evidence that OH stretching frequencies are sensitive to the local electric field experienced by the hydrogen atoms in these clusters. The magnitude of the Stark shifts calculated for these clusters is greater than that of the experimental data. For example, the experimental frequency shift of the AAD band in La^3+^(H_2_O)_*n*_ clusters between *n* = 20 and 300 is ∼30 cm^–1^ whereas the calculated shift for this size range is ∼60 cm^–1^. Overall, the calculated frequencies agree fairly closely with the experimental frequencies and on average deviate by ∼14 cm^–1^. Extrapolations of the IRPD data to infinite cluster size give free OH stretch frequencies centered on ∼3698 cm^–1^ whereas the modeled data is centered on ∼3715 cm^–1^. Thus, this simple electrostatic model can account for the major experimental trends in Stark shifting with ion charge state.

There are also significant differences between the simulated and experimental Stark shifting data. For Mo^3+^ and Ca^2+^, the calculated Stark shifting is overall more linear as a function of 1/*r* ^2^ than what is observed experimentally and do not replicate the more pronounced Stark shifting at small cluster size. This suggests that the models used in these simulations do not accurately account for the ion-induced orientations of water molecules at the surfaces of the smaller droplets. Additionally, the slope of the simulated Na^+^(H_2_O)_*n*_ data is opposite in sign to what is observed in experiment, and the computed slope of the neutral nanodrops is significantly lower than that inferred from the experimental data. This indicates that the simulations do not accurately reproduce the effect of the electric field attributed to the orientation of water at the surface of these nanodrops. The limitations of the point charge model used to simulate infrared spectra have been described in detail elsewhere.^82^ Briefly, because all atoms are considered as fixed point charges, neither charge transfer nor polarization effects are accounted for. Although this model includes the effects of intramolecular coupling between OH stretches, it does not explicitly include intramolecular coupling to the H_2_O bend or intermolecular coupling between water molecules. The insights gained from this model, despite its shortcomings, call for the development of more sophisticated electrostatic models that take into account vibrational coupling/polarization effects.

### Hydration motifs and droplet composition

**Fig. 7:**
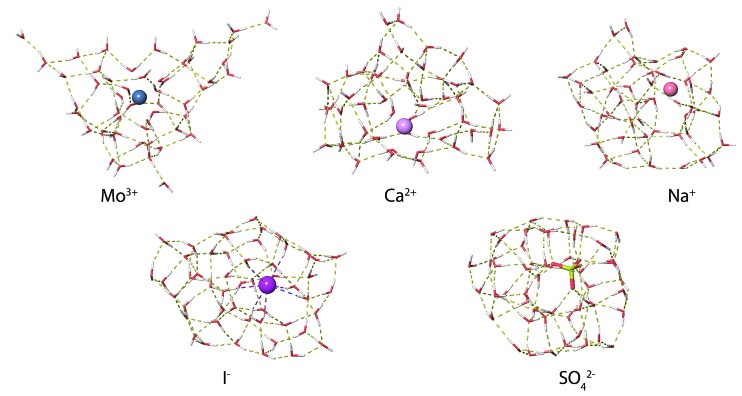
Representative low-energy structures of M(H_2_O)_50_ where M = Mo^3+^, Ca^2+^, Na^+^, Li^+^, I^–^ and SO_4_^2–^ identified from molecular dynamics simulations at 133 K.

MD simulations were used to investigate how ions affect the H-bonding network of water in aqueous nanodrops. The lowest-energy structures identified from MD simulations at 133 K for M(H_2_O)_50_ where M = Mo^3+^, Ca^2+^, Na^+^, Li^+^, I^–^ and SO_4_^2–^ show significant differences in ion hydration motifs (Fig. 7). Many low-energy structures of each nanodrop are present in these experiments, and although limited conclusions can be drawn from a single computed structure, comparisons between these structures illustrate general trends in hydration motifs as the ion charge and polarity are varied. The structure of Mo^3+^(H_2_O)_50_ is highly puckered due to strong Mo^3+^–water interactions that result in under-coordinated water molecules at the surface of the cluster. This suggests that multiply charged ions can disrupt optimal H-bonding between water molecules. As the charge state of the solvated cation decreases, so too does the ion's effect on the solvent. The calculated structures of Ca^2+^(H_2_O)_50_ and Na^+^(H_2_O)_50_ have progressively fewer under-coordinated water molecules, with the latter structure being more compact in shape as H-bonding between water molecules is better optimized in clusters with lower charge. Fundamentally different hydration motifs are apparent in the structures of the anions I^–^(H_2_O)_50_ and SO_4_^2–^(H_2_O)_50_ where OH bonds are directed inwards towards the solvated ion resulting in fewer free OH bonds at the surfaces of the clusters and more optimized water–water H-bonding. The small number of free OH oscillators for SO_4_^2–^(H_2_O)_50_ is consistent with the very weak free OH band in the IRPD spectrum of this cluster. Additionally, the tangential orientation of these free OH bonds with respect to the cluster surface is consistent with a decrease in the magnitude of Stark shifting for smaller SO_4_^2–^(H_2_O)_*n*_ clusters on account of poorer overlap with the ion's electric field.

A more comprehensive view of how nanodrop composition changes with ion charge state and extent of hydration for M(H_2_O)_*n*_ where M = Mo^3+^, Ca^2+^, Na^+^, I^–^, and SO_4_^2–^ and *n* = 50, 100, and 250 is given in Table 1, which shows the relative populations of different types free OH stretches taken from averages over 1000 calculated structures. Free OH stretches are categorized according to the H-bonding environment of the associated water molecule as either AAD, AD, or “UC” where the last category encompasses all types of under-coordinated water molecules (A, AA, D). There are two general trends in these data. First, for a fixed cluster size, the droplets containing high valency ions have the greatest percentage of under-coordinated surface water molecules. Second, for a given ion, as the droplet size increases, H-bonding at the surface of the cluster becomes more optimal as indicated by the greater percentage of AAD and AD stretches. This trend is most striking for the Mo^3+^(H_2_O)_*n*_ hydrates where for *n* = 50, nearly 30% of free OH stretches arise from dangling A and AA water molecules. As the size of the nanodrop increases, the solvated ion's influence on the surface structure diminishes and by *n* = 250, there are virtually no under-coordinated water molecules. This is qualitatively consistent with the IRPD spectra of La^3+^(H_2_O)_*n*_ insofar as the spectra of smaller cluster sizes (*n* ≤ 38) have A and AA bands that are not observed in the spectra of the larger clusters. We note that the populations of different free OH stretches taken from these calculations cannot be used to directly predict band intensities because the transition dipole moments of the stretches are not taken into account. For the anionic clusters, the hydration motif is reversed and even at the smallest cluster size (*n* = 50), there are no under-coordinated water molecules. Yet the surfaces are still strained; SO_4_^2–^(H_2_O)_50_ has on average only 1.1 free OH bonds owing to the strong effect of SO_4_^2–^ on surface water molecules. For both cationic and anionic clusters with 250 water molecules, the relative populations of AAD and AD stretches become nearly equal as the effect of an ion on nanodrop surface structure diminishes. Interestingly, although the H-bonding environments of free OH stretches are similar at *n* = 250, the percentage of OH groups that are free OH stretches still depends upon the identity of the ion (Mo^3+^: 11%, Ca^2+^: 9%, Na^+^: 8%, I^–^: 8%, SO_4_^2–^: 4%) consistent with a previous IRPD study that compared band intensities in detail at this cluster size.^27^ These results provide further evidence that ion–water interactions established in the interior of the nanodrop give rise to hydration patterns that propagate out to the nanodrop's surface.

**Table 1:** Relative populations of different types of free OH stretches in calculated structures of M(H_2_O)_*n*_ where M = Mo^3+^, Ca^2+^, Na^+^, I^–^, and SO_4_^2–^ for *n* = 50, 100, and 250. The category of stretches labeled as “UC” includes A, AA, and D stretches arising from under-coordinated water molecules

	Mo^3+^	Ca^2+^	Na^+^	I^–^	SO_4_^2–^
**M(H**_**2**_ **O)**_**50**_					
% AAD	61	76	76	93	43
% AD	10	17	24	7	57
% UC	29	7	0	0	0

**M(H**_**2**_ **O)**_**100**_					
% AAD	71	84	89	87	59
% AD	9	15	11	13	41
% UC	20	1	0	0	0

**M(H**_**2**_ **O)**_**250**_					
% AAD	85	84	86	88	78
% AD	15	16	14	12	22
% UC	0	0	0	0	0

**Fig. 8:**
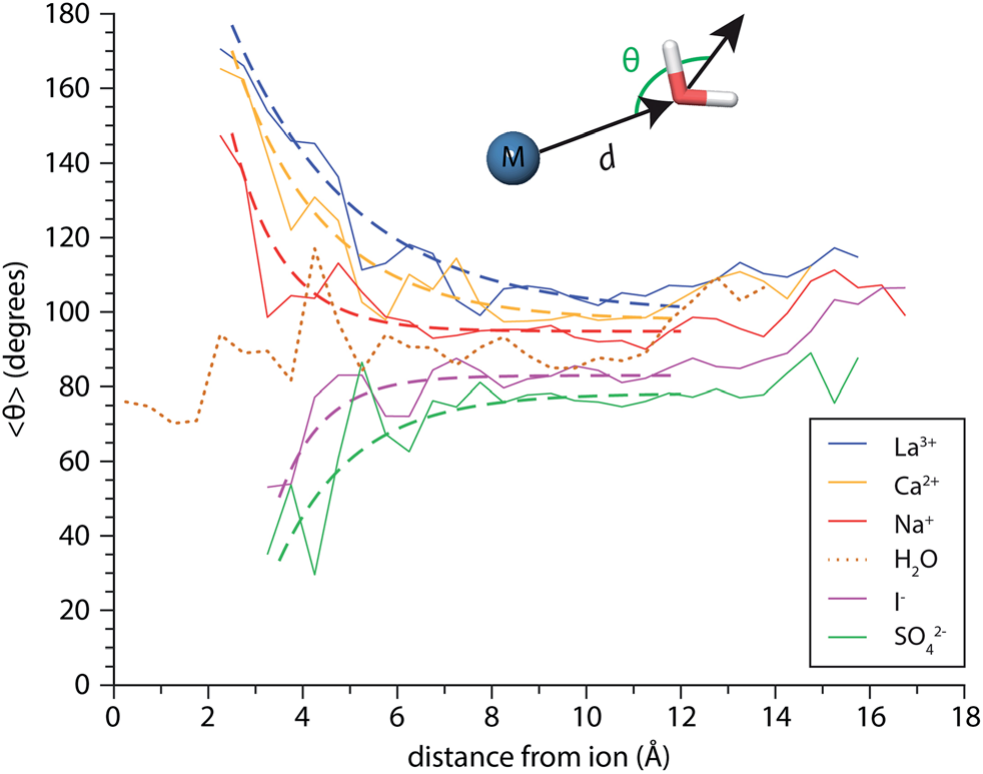
Average angular orientation of the water molecule dipole (*θ*) as a function of distance *d* from the ion calculated from MD simulations of M(H_2_O)_250_ where M = Mo^3+^, Ca^2+^, Na^+^, I^–^, and SO_4_^2–^ binned in 0.5 Å increments. The dashed lines are exponential fits to these data for *d* ≤ 12 Å. Results from simulations of (H_2_O)_250_ are shown as a dotted brown line, and distances are reported with respect to the center-of-mass of these clusters.

Perturbations of surface water structure must ultimately arise from the effects of solvated ions on the H-bonding network of water in the interior of the nanodrop. Information about the extent of ion-induced patterning was estimated from MD simulations of the *n* = 250 clusters for Mo^3+^, Ca^2+^, Na^+^, I^–^, and SO_4_^2–^, as well as (H_2_O)_250_ clusters. The average orientations of the dipole moments of water molecules, *θ*, are shown a function of distance *d* from the ion in Fig. 8 as solid lines. For the neutral water clusters, the orientation data is plotted as a function of distance from the center-of-mass of the cluster. These data show that the orientations of water molecules within the nanodrop are strongly influenced by both ion charge state and polarity. In the cationic nanodrops, water molecules are oriented “outwards” away from the ion and this patterning effect is strongest for water molecules located closest to the ion. Similar trends are observed for the anions, but in these nanodrops, water molecules are oriented “inwards” such that the OH bonds point towards the anion. For the neutral nanodrops, the orientations of water molecules in the interior of the droplet are more randomly distributed. In order to characterize the spatial extent of ion-induced patterning within the nanodrops, these data were fit with exponential functions for *d* ≤ 12 Å (dashed lines), where the cutoff distance was chosen to exclude surface effects on water orientation.^55^ The fits of the Na^+^ and I^–^ data indicate that the influence of these ions on the orientations of water molecules decays quickly with distance and is weak beyond distances corresponding to the first hydration shell, consistent with reports that these ions minimally perturb the structure of water.^27,82^ In contrast, the patterning effect for Mo^3+^ as well as Ca^2+^ and SO_4_^2–^ is significantly stronger and can extend up to ∼1 nm into the nanodrop. These findings are consistent with changes in the magnitude of the Stark shifting arising from a solvent patterning effect. For all of the ions, the simulations suggest that hydration motifs established around the solvated ion can propagate out to the nanodrop's surface.

## Conclusions

The confined environments of aqueous nanodrops serve as ideal systems in which to study the effect of a single ion on the H-bond network of water. Structural changes in size- and charge-selected nanodrops were investigated with IRPD spectroscopy on M(H_2_O)_*n*_ clusters for M = La^3+^, Ca^2+^, Na^+^, Li^+^, I^–^, and SO_4_^2–^ with up to 550 water molecules attached. Spectral signatures in the free OH region originating from surface-bound water molecules show that multiply charged ions can significantly influence hydration motifs in these nanodrops. For hydrates of La^3+^ and Ca^2+^, there are bands corresponding to under-coordinated A and AA water molecules in spectra of the smaller clusters (*n* < ∼40) indicating that strong ion–water interactions disrupt optimal H-bonding between water molecules. Intrinsically different interactions between anions and water molecules leads to the suppression of free OH stretches in the smaller anionic clusters. The dependence of nanodrop structure on ion charge state and polarity is supported by molecular dynamics simulations, which indicate that hydration motifs established around the ion in the interior of the nanodrop can propagate to the nanodrop's surface even for distances exceeding 1 nm.

The frequency of the AAD band in the IRPD spectra of these clusters depends upon ion charge state, ion polarity, ion size and droplet size. These data are consistent with a Stark shift from the electric field of the ion as well as that of water itself at the surfaces of the droplets. The frequency of the AAD band extrapolated from the larger clusters to infinite droplet size and thus infinite dilution gives a value between 3696.5–3701.0 cm^–1^. Stark shifting of the free OH band in nanodrops with more than ∼100 water molecules depends primarily on the electric field of the solvated ion and not on ion-induced patterning effects. The IRPD spectra indicate that the surface structure of water molecules in these larger nanodrops is similar and should approach that of bulk water with increasing cluster size. Thus, these measurements provide the most precise assignment of the free OH stretching frequency at the surface of liquid water.

The experimental Stark shifting data exhibit varying degrees of non-linearity that is more pronounced for ions with a greater charge density and are best fit by two lines. This effect is especially prominent for smaller La^3+^(H_2_O)_*n*_ and Ca^2+^(H_2_O)_*n*_ clusters, where the slope of Stark shifting is greater in clusters with less than ∼100 water molecules. We attribute this effect to the polarization of water molecules surrounding the solvated ion, which leads to structural changes at the nanodrop surface. Multiply charged cations such a La^3+^ and Ca^2+^ can align surface free OH bonds along their electric field lines, resulting in more pronounced Stark shifting at small cluster size where this orientation effect is the strongest. These data suggest that multiply charged cations can affect the H-bond network of water molecules at distances corresponding to at least the third hydration shell. This long-range pattering effect has previously been demonstrated for a variety of multiply charged anions where the appearance of a free OH band at large cluster size indicates the weakening of ion–water interactions. For cations, free OH bonds are intrinsically directed away from the ion even at small cluster size and so the appearance of this band cannot be used to deduce solvent patterning effects. The pronounced changes in Stark shifting for cations reported here provide a distinct spectroscopic signature for the spatial extent of ion–water interactions in these nanodrops.

Free OH bands red-shift in aqueous nanodrops containing multiply charged cations and blue-shift in nanodrops containing anions, and this trend is reproduced by a computationally inexpensive electrostatic model for simulating infrared spectra of these clusters. However, this model does not reproduce data for 1+ ions and neutral droplets. A small blue shift of the free OH bands in IRPD spectra of Na^+^(H_2_O)_*n*_ and Li^+^(H_2_O)_*n*_ clusters with decreasing cluster size despite the positive charge of the ions suggests that the intrinsic structure of water in small nanodrops generates a surface electric field similar to anion-containing clusters. The interpolated Stark shifts for neutral water clusters indicate that the surface potential of water depends on cluster size, becoming more positive with decreasing cluster size. To our knowledge, this is the first evidence that water clusters containing tens to hundreds of water molecules have established surface potentials, which is a topic that has recently been debated.^99^

These findings provide new insights into ion hydration and the structure of water in confined environments, which are important for a variety of physical properties including Hofmeister effects. The experimental results also provide stringent benchmarks for theoretical modeling of ion hydration, and our limited success in using simple electrostatic models to simulate Stark shifting in these clusters calls for the development of more sophisticated electrostatic models that can reproduce phenomena related to ion solvation with greater accuracy.
