# Supplementary material for: Structural and electrostatic effects at the surfaces of size- and charge-selected aqueous nanodrops
Source: Chem Sci. 2017 May 19;8(7):5201–13. doi: 10.1039/c7sc00481h (PMC5618692; doi:10.1039/c7sc00481h)
Supplement: Supplementary file 1 [file SC-008-C7SC00481H-s001.pdf]

Electronic Supplementary Material (ESI) for Chemical Science.
This journal is © The Royal Society of Chemistry 2017

Structural and Electrostatic Effects at the Surfaces of Size- and Charge-Selected Aqueous Nanodrops

Richard J. Cooper, Jeremy T. O'Brien, Terrence M. Chang and Evan R. Williams*

Department of Chemistry, University of California, Berkeley, California, 94720-1460

Supplementary Information

	La^{3+}	Ca^{2+}	Na^{+}	Li^{+}	I^{-}	SO_4^{2-}
**Fit 1 – small**						
cluster sizes (*n*)	20-102	20-100	20-30	20-36	-	50-80
slope	-313.0	-132.8	-21.8	-50.3	-	59.8
intercept (cm^{-1})	3708.0	3703.8	3704.1	3703.5	-	3706.1
**Fit 2 - large**						
cluster sizes (*n*)	102-550	100-300	30-250	36-120	20-250	80-300
slope	-66.1	4.1	51.2	8.4	111.6	130.1
intercept (cm^{-1})	3696.5	3697.5	3696.5	3698.2	3699.4	3701.0

Table S1. Calculated slopes and intercepts from linear fits of the Stark shifting data in Figure 5 at both small and large cluster sizes.

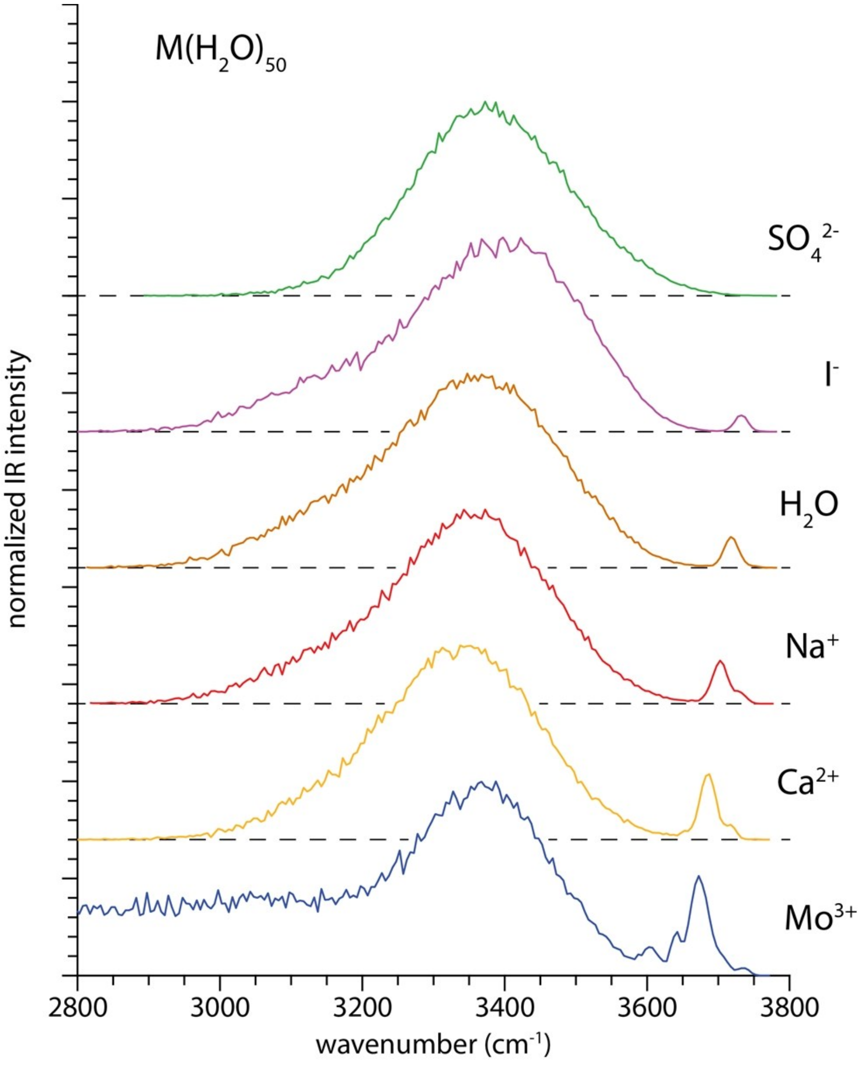

Figure S1. Calculated infrared spectra of $(H_2O)_{50}$ and $M(H_2O)_{50}$ where M = Mo^{3+}, Ca^{2+}, Na^{+}, I^{-} and SO_4^{2-}. Spectra are calculated from 1000 structures identified by MD simulations using an electrostatic point charge model that accounts for intramolecular coupling and frequency-dependent transition intensities. The sharp bands between 3600–3800 cm^{-1} arise from free OH stretches at the surfaces of the nanodrops whereas the broad resonances at lower energy correspond to hydrogen bonded OH stretches throughout the nanodrops.
